# Analysis of immune related gene expression profiles and immune cell components in patients with Barrett esophagus

**DOI:** 10.1038/s41598-022-13200-6

**Published:** 2022-06-02

**Authors:** Lin Shi, Renwei Guo, Zhuo Chen, Ruonan Jiao, Shuangshuang Zhang, Xuanxuan Xiong

**Affiliations:** Department of Gastroenterology, Xuzhou Central Hospital, Xuzhou, Jiangsu China

**Keywords:** Cancer, Computational biology and bioinformatics, Genetics, Immunology

## Abstract

Barrett's esophagus (BE) is a well-known precancerous condition of esophageal adenocarcinoma. However, the immune cells and immune related genes involved in BE development and progression are not fully understood. Therefore, our study attempted to investigate the roles of immune cells and immune related genes in BE patients. The raw gene expression data were downloaded from the GEO database. The limma package in R was used to screen differentially expressed genes (DEGs). Then we performed the least absolute shrinkage and selection operator (LASSO) and random forest (RF) analyses to screen key genes. The proportion of infiltrated immune cells was evaluated using the CIBERSORT algorithm between BE and normal esophagus (NE) samples. The spearman index was used to show the correlations of immune genes and immune cells. Receiver operating characteristic (ROC) curves were used to assess the diagnostic value of key genes in BE. A total of 103 differentially expressed immune-related genes were identified between BE samples and normal samples. Then, 7 genes (CD1A, LTF, FABP4, PGC, TCF7L2, INSR,SEMA3C) were obtained after Lasso analysis and RF modeling. CIBERSORT analysis revealed that resting CD4 T memory cells and gamma delta T cells were present at significantly lower levels in BE samples. Moreover, plasma cell and regulatory T cells were present at significantly higher levels in BE samples than in NE samples. INSR had the highest AUC values in ROC analysis. We identified 7 immune related genes and 4 different immune cells in our study, that may play vital roles in the occurrence and development of BE. Our findings improve the understanding of the molecular mechanisms of BE.

## Introduction

Barrett's esophagus (BE) is a well-documented precancerous condition of esophageal adenocarcinoma (EAC)^1^, which is characterized by the occurrence of metaplasia and is followed by cellular changes in the columnar epithelium^2^ due to many causes, such as gastroesophageal reflux disease (GERD)^3^. It is difficult to define an accurate global prevalence of BE However, recent studies have shown that nearly 15% of GERD patients suffer from BE worldwide and the incidence of BE is higher in patients with heartburn^2^. A multinational systematic review of 28 studies reported that the prevalence of GERD in adult populations had increased by approximately 50% in 20 years^4^. As a common complication of GERD, the incidence of BE is rapidly increasing^3^. Therefore , it can be confirmed is that the overall prevalence of BE is increasing^5^. However, the molecular mechanism of BE is still unclear.

The local immune system in esophageal mucous plays a vital role in BE development and progression^6,7^. Factor fork head box protein P3 (FOXP3) is always a marker of CD4 + regulatory T cells, which suppress local immunity, aid in tumor cell immune escape and promote tumor development and progression^8^. Previous studies showed that BE tissues always had significantly higher FOXP3 expression than normal tissues^9^. Moreover, FOXP3 + T cells were more common in BE tissues^10^. In addition, study also shown that RALDH2, an anti-inflammatory gene, was associated with the expression of myeloid dendritic cells and had a higher expression in BE tissues^9^.

Although immune cells have been evaluated in many studies, the impact of immune cells and immune related genes in BE development and progression has not been fully investigated. Fortunately, bioinformatics analysis generates large and complex biological data, and these biological data can help us study the molecular mechanisms of different diseases^11,12^. Therefore, our present study aims to analyze immune-related gene expression profiles and immune infiltration in BE patients with bioinformatic methods. Our study will provide new insights into the pathogenesis of BE and may help develop immunotherapies for BE patients.

## Materials and methods

### Data collection

The transcription profile dataset of Barrett's Esophagus was obtained from the NCBI GEO databases (http://www.ncbi.nlm.nih.gov/geo/). The accession number is GSE39491^13^, which is based on the GPL571 Platform. The dataset contains 80 fresh frozen tissue samples of Barrett’s metaplasia (40 samples) and matched normal esophagus (NE, 40 samples) from squamous esophagus. The background correction, normalization and probe summarization of the microarray dataset with raw data were carried out by R software. The 2498 immune-related genes were downloaded from the ImmPort database (Immunology Database and Analysis Portal database, https://www.immport.org/shared/home).

### Differentially expressed immune-related gene identification

The Linear Models for Microarray Data (limma) package in Bioconductor was used to identify differentially expressed genes (DEGs) by comparing expression values between Barrett’s metaplasia and normal mucosa. Genes with cutoff criteria of |log2FC (fold change)|> 1 and adjusted p value < 0.05 were selected as the threshold for DEGs. We used Benjamini–Hochberg procedure, also known as FDR method, to adjust the p value^14,15^. A volcano plot and a heatmap were used to display these genes. Then, the DEGs were overlapped with the 2498 immune-related genes, resulting in 103 immune-related DEGs.

### GO and KEGG enrichment analyses of DEGs

Using the clusterProfiler R package from Bioconductor, we performed Gene Ontology analysis and Kyoto Encyclopedia of Genes and Genomes (KEGG) pathway analysis to identify the biological processes for 103 immune-related genes^16–18^. P < 0.05 was considered as the cut‐off criterion.

### Identification of key genes by lasso and random forest analysis

By constructing a penalty function for all variables, Lasso can compress unimportant variable coefficients to 0, thus excluding those variables, and then the independent variables that have a greater impact on the outcome are selected in the final analysis. The 103 immune-related genes were entered into the Lasso regression analysis to screen key genes by the glmnet package in R. We also constructed a random forest model (RF) to screen key genes by the randomForest package in R^19,20^. RF is an algorithm that performs classification or regression by combining the voting results of multiple decision trees. The number of decision trees constructed in this study was 500. The RF selected or excluded variables according to the feature importance. An RF model was used to predict the BE status in each sample based on gene expression profiles. Mean decrease accuracy is an important indicator of variable importance, which directly measures the effect of each variable^21^. Therefore, mean decrease accuracy was used to identify core genes. In our study, we refer to some previous studies that the results of mean decrease gini were similar to mean decrease accuracy^22^. Therefore we also show mean decrease gini at the same time. The top 20 genes with mean decreased accuracy were selected as key genes by the RF model^19,22^. The over lapping genes after LASSO and RF analyses were used as the key genes.

### Analysis of immune cell infiltration

Using its deconvolution algorithm, CIBERSORT can quantify the abundance levels of 22 immune cell subtypes based on the expression files^23,24^. To compare the difference between BE samples and NE samples in immune cells, the CIBERSORT package was used in R software. Samples with P < 0.05 in the CIBERSORT analysis results were used in further analysis. The Mann–Whitney U test was used to compare differences in immune cell subtypes, and violin plots were generated for the BE samples and NE samples.

### Statistical analysis

The Wilcox test was used to compare the proportions of 22 immune cells based on CIBERSORT analysis between BE samples and NE samples. ROC curves were used to evaluate the diagnostic value of each core gene and were constructed by Stata 14.0. P < 0.05 was considered statistically significant.

## Results

### Identification of DEGs and immune related DEGs

**Figure 1:**
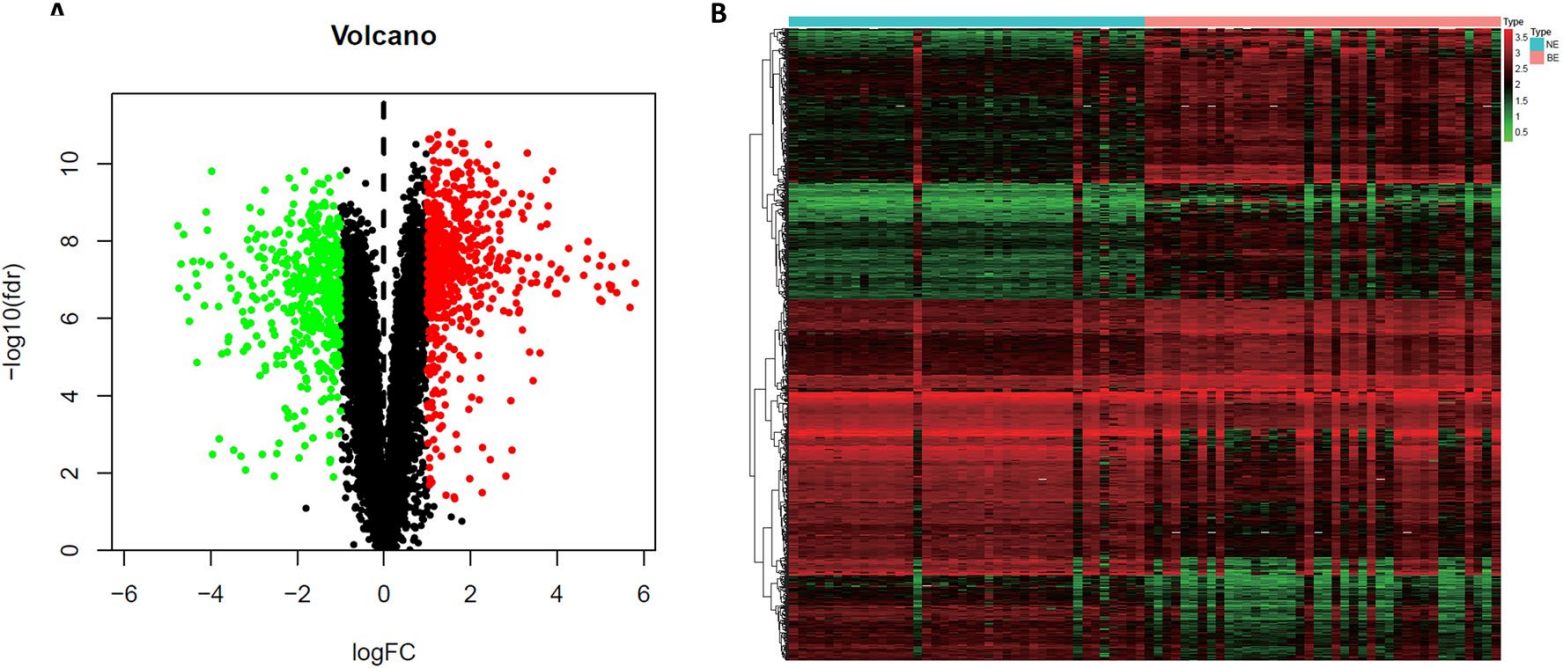
Identification of DEGs form GEO dataset. (**A**) The volcano plot of DEGs between BE and NE samples. (**B**) The heatmap plot of DEGs between BE and NE samples.

**Figure 2:**
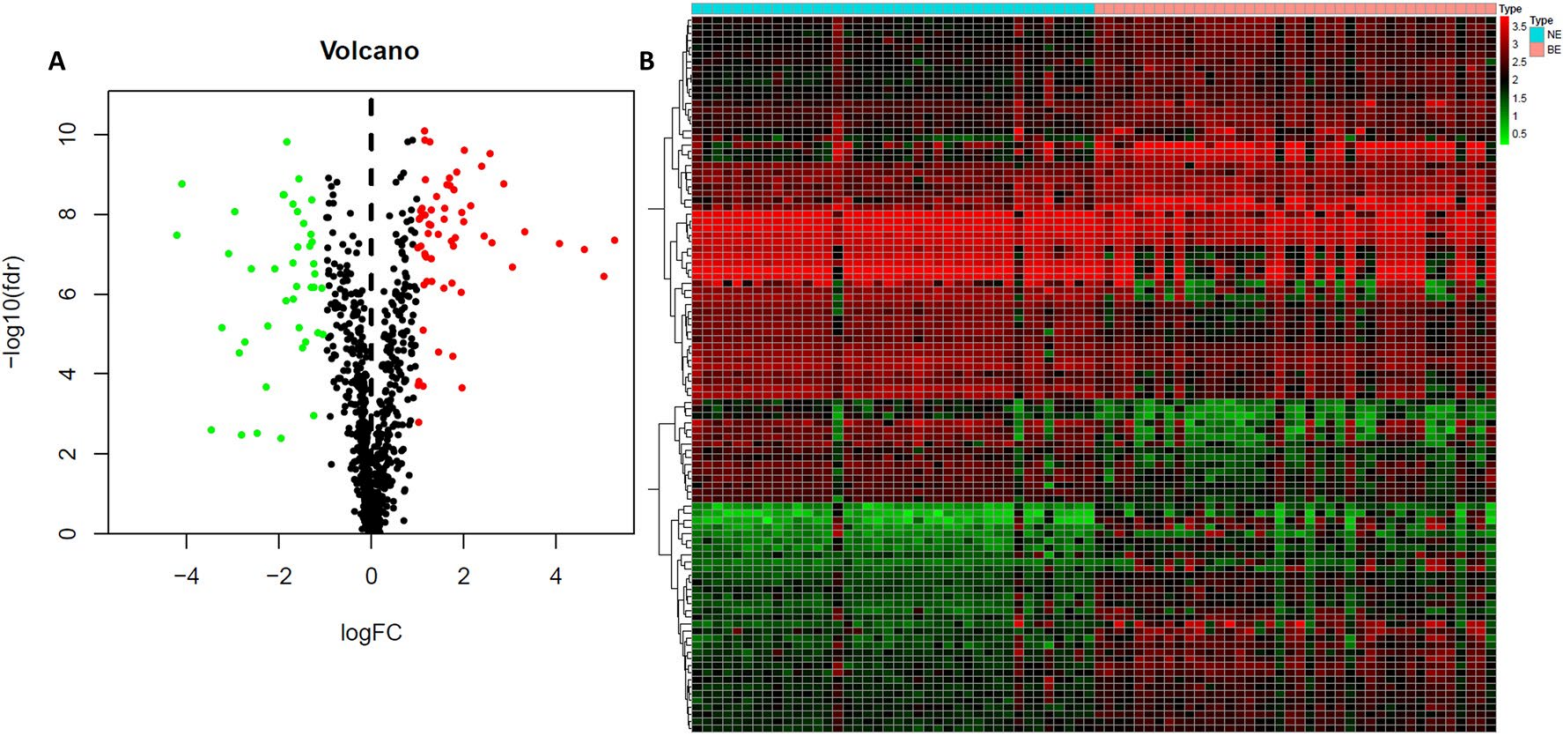
Identification of DEGs form GEO dataset. (**A**) The volcano plot of immune related DEGs between BE and NE samples. (**B**) The heatmap plot of immune related DEGs between BE and NE samples.

After data preprocessing, 1121 DEGs were screened between BE and NE samples, including 480 upregulated genes and 647 downregulated genes (Supplementary table 1). The distribution and expression of DEGs are shown by volcano plot and heatmap plot (Fig. 1). The 2498 immune-related genes overlapped with 1121 DEGs, obtaining 103 immune related genes. A volcano plot and a heatmap plot of overlapping immune-related genes are also shown (Fig. 2A,B, Supplementary table 2).

### Enrichment analysis for immune-related DEGs

**Figure 3:**
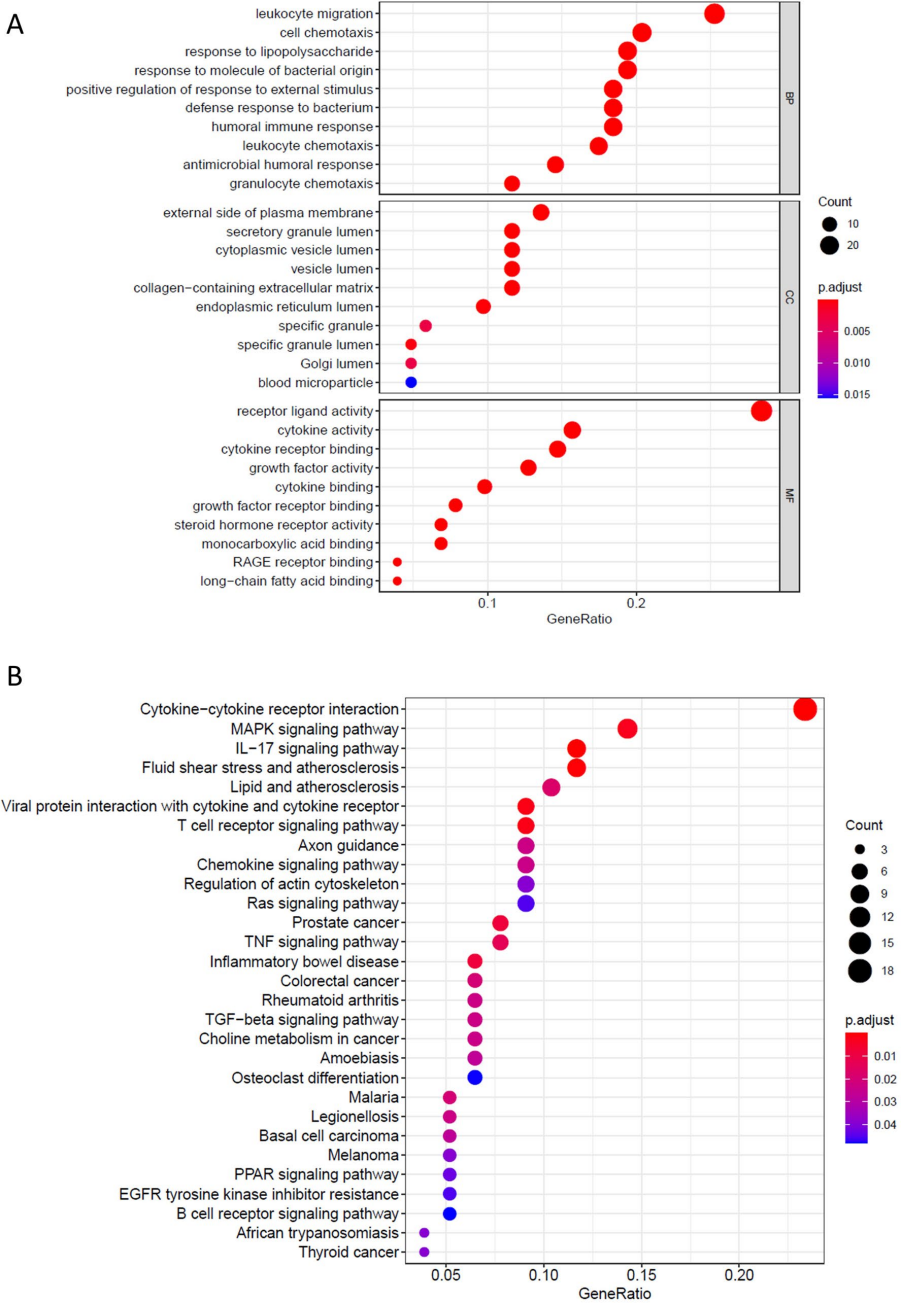
GO function analysis and KEGG pathway analysis. (**A**) GO functional enrichment analysis of the immune-related DEGs. (**B**) KEGG functional enrichment analysis of the immune-related DEGs.

GO and KEGG analyses were performed to further explore the mechanisms of immune-related DEGs. The top 5 GO terms were receptor ligand activity, leukocyte migration, cell chemotaxis, response to lipopolysaccharide and response to molecules of bacterial origin (Fig. 3A). In addition, the KEGG items were associated with cytokine − cytokine receptor interaction, MAPK signaling pathway, IL − 17 signaling pathway, fluid shear stress and atherosclerosis and lipid and atherosclerosis (Fig. 3B).

### Identification of key immune-related DEGs by LASSO analysis

**Figure 4:**
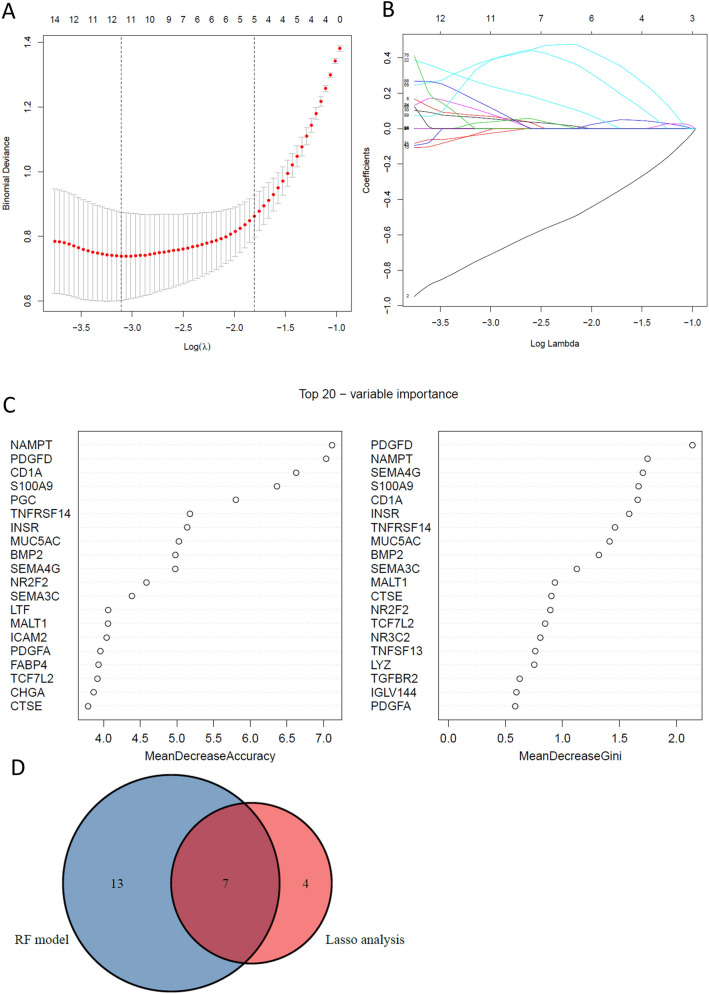
Identification of the optimal immune-related biomarkers. (**A**,**B**) LASSO regression analysis. (**C**) Top 20 genes by RF model sort by accuracy. (**D**) Venn diagram of overlapping.

To further identify key immune-related genes, LASSO analysis was performed for the 103 immune-related DEGs. Eleven candidate key genes were identified by LASSO analysis: CD1A, CXCL14, LTF, FABP4, PGC, MUC4, TCF7L2, SEMA3C, IL1R2, INSR, and IL12A, which were considered candidate optimal immune related biomarkers (Fig. 4A,B). In addition, the RF for the 103 immune-related DEGs was also used to screen gene signatures, and the top 20 gene signatures were retrieved (Fig. 4C, Supplementary table 3). Ultimately, there were 7 key overlapping genes after LASSO and RF analyses, CD1A, LTF, FABP4, PGC, TCF7L2, INSR, and SEMA3C, which were considered as the optimal immune-related biomarkers (Fig. 4D).

### Immune cell analysis between BE and NE samples

**Figure 5:**
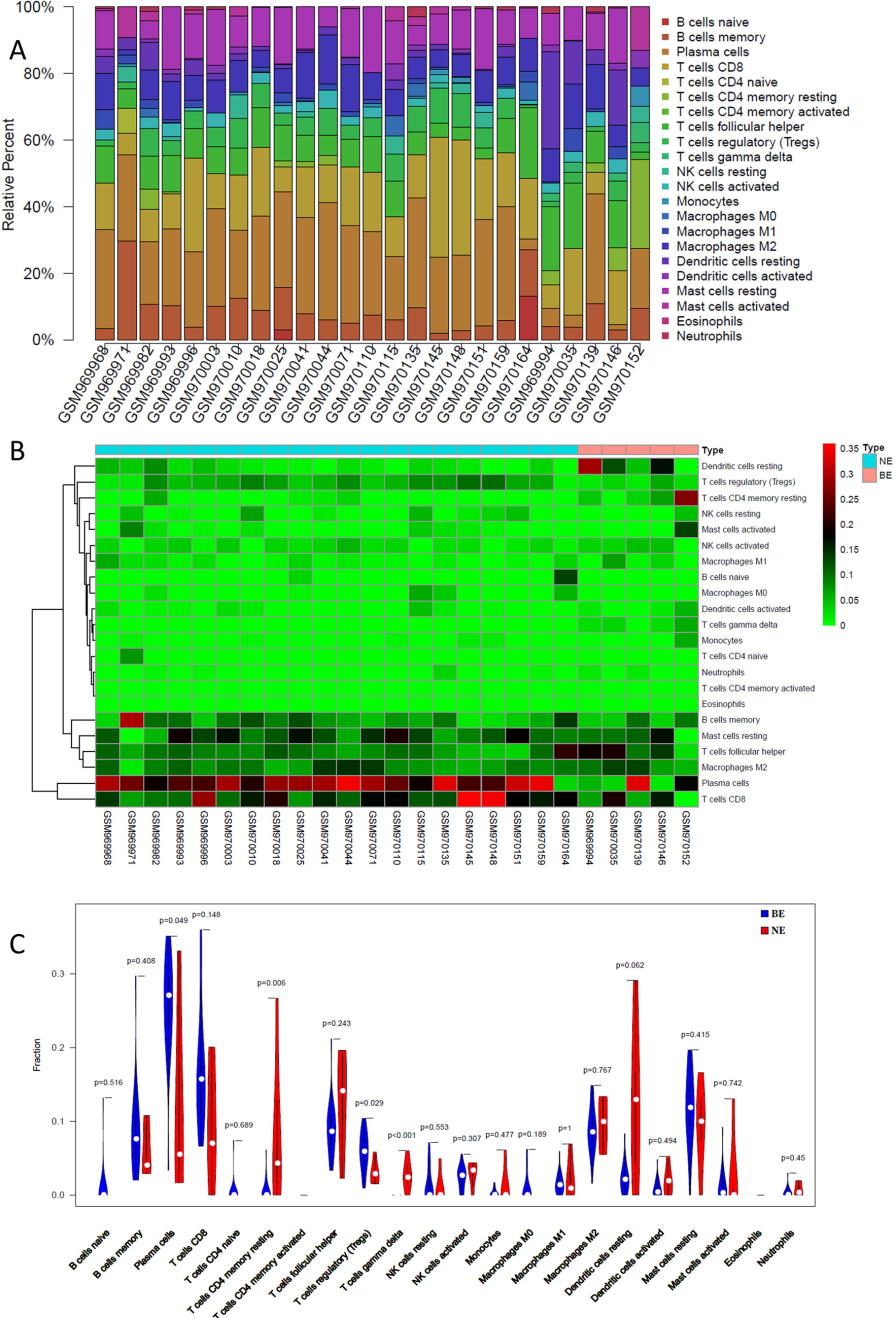
Distribution of immune cells between BE and NE samples. (**A**) Percentage of immune cells in each sample. (**B**) Heatmap. (**C**) Violin plot.

The abundance levels of the 22 immune cells between BE samples and NE samples were further analyzed. The CIBERSORT algorithm indicated that plasma cells, CD8 + T cells, gamma delta T cells, and resting mast cells had a larger proportions in the BE and NE samples (Fig. 5A). A heatmap was drawn to show the proportions of immune cells in each sample (Fig. 5B ). Figure 5C shows that resting CD4 T memory cells (p = 0.006), and gamma delta T cells (p < 0.001) were significantly lower in BE samples. In addition, plasma cells (p = 0.049) and regulatory T cells (p = 0.029) were significantly higher in BE samples than in NE samples. Differences in immune cells suggested the important role of the immune system in Barrett's esophagus.

### Correlation analysis between key genes and immune cells

**Figure 6:**
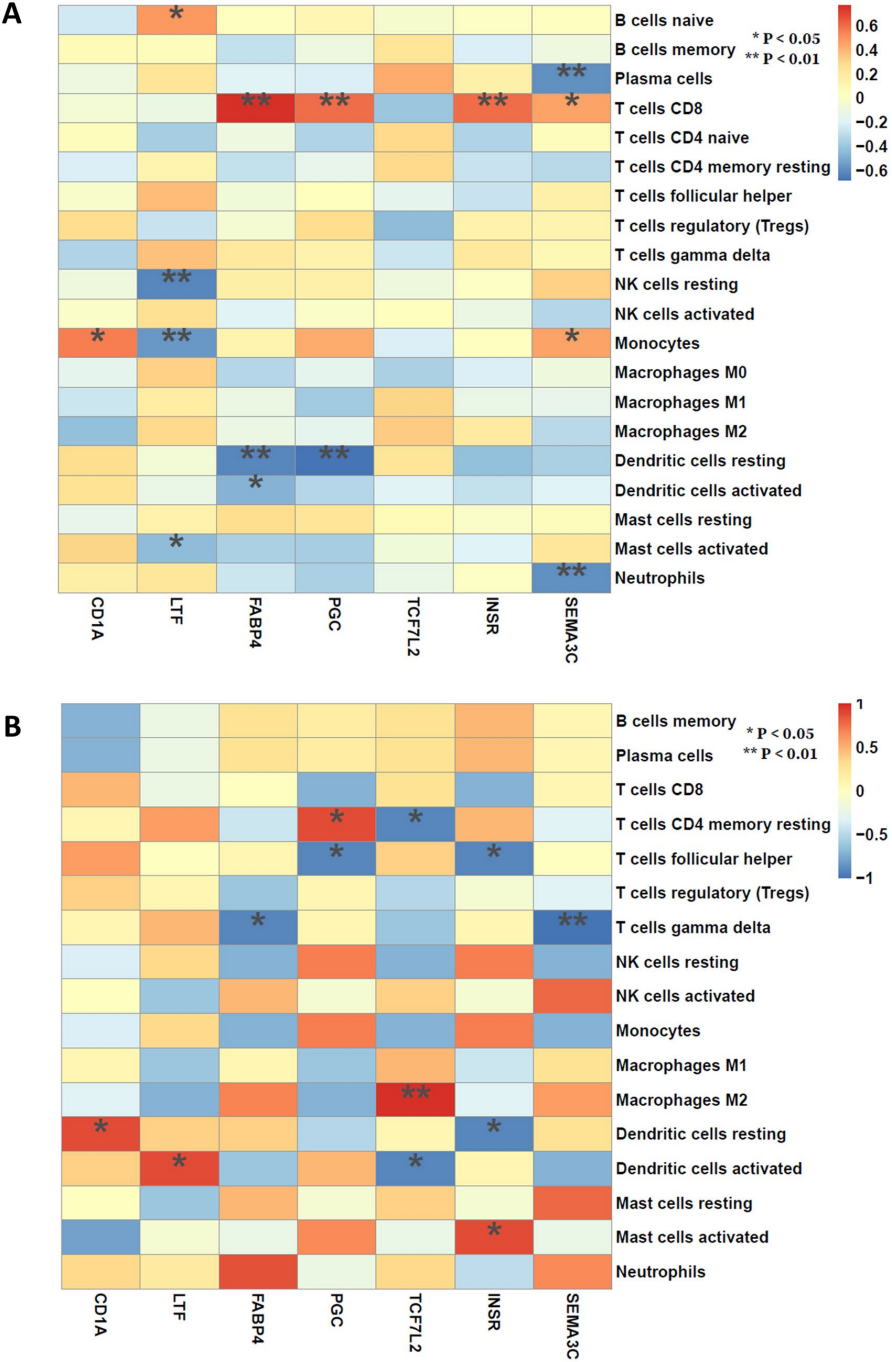
Spearman correlation of the key genes and the immune cells. (**A**) Correlation in BE samples. (**B**) Correlation in NE samples.

A Spearman correlation heatmap was drawn to show the relevance between key genes and immune cells among BE and NE samples. CD1A was only significantly associated with monocytes and resting dendritic cells in BE samples and NE samples, respectively. In BE samples, the LTF gene was associated with naïve B cells, resting NK cells and monocytes ,whileFABP4 was also associated with CD8 + T cells, and resting and activated dendritic cells. The PGC gene was associated with CD8 + T cells and resting dendritic cells. Moreover, INSR was significantly associated with CD8 + T cells. SEMA3C was significantly associated with CD8 + T cells, monocytes, plasma cells and neutrophils. Additionally, there were significant differences between T1DM and normal samples; the details are shown in the heatmap (Fig. 6A,B).

### ROC analysis

**Figure 7:**
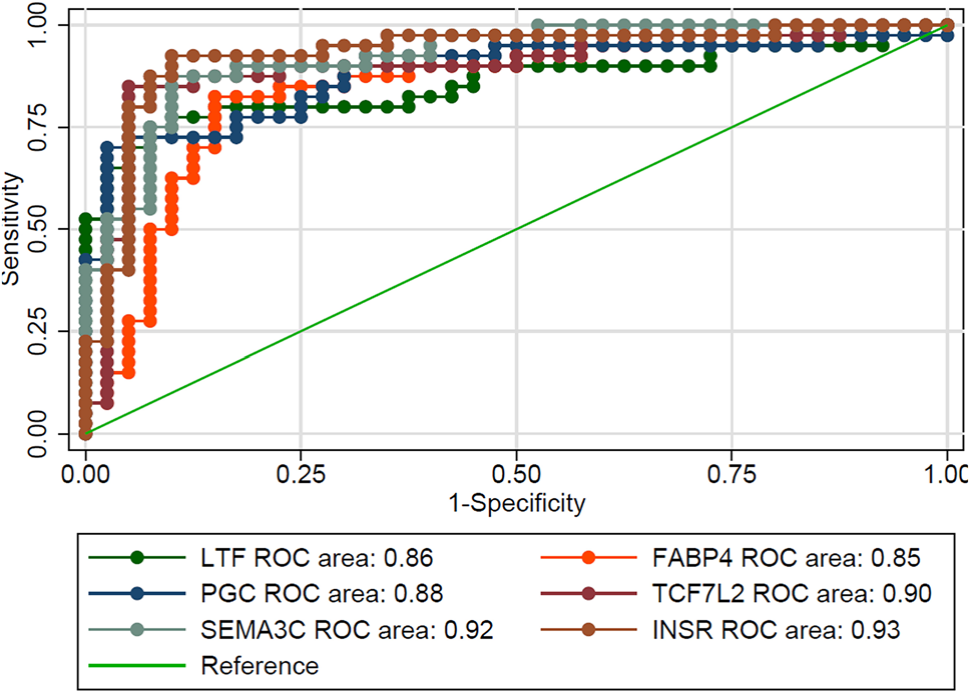
The ROC curve of each key genes.

ROC curves were performed to further explore the diagnostic accuracy of key genes respectively. Genes with an area under the ROC curve greater than 0.8 are shown in the article with INSR having the highest AUC values (Fig. 7).

## Discussion

BE is a very common esophageal mucosal lesion worldwide. Immune cell components and immune related genes are vital in the BE and EAC microenvironments. In the present study, we identified 103 immune related DEGs between BE and NE samples based on genomic expression profile analysis. Then 11 candidate immune-related key biomarkers for BE were obtained by LASSO analysis, and the RF model was also used to screen potential key genes. Then, the 7 overlapping genes (CD1A, LTF, FABP4, PGC, TCF7L2, INSR, SEMA3C) from LASSO analysis and the RF model used to select the key genes. In addition, the CIBERSORT algorithm application indicated that CD4 T memory cells and gamma delta T cells were present at significantly lower levels in BE samples. Plasma cell and regulatory T cells were present at significantly higher levels in BE samples than in NE samples. Furthermore, we analyzed the correlation between key biomarkers and immune cells.

We first performed differential gene analysis by R software and obtained 1121 DEGs, Then103 immune-related genes involved in BE were identified by intersecting 2498 immune-related genes with 1121 DEGs. Furthermore, enrichment analysis for immune related DEGs was conducted to explore gene functions. KEGG analysis indicated that cytokine—cytokine receptor interaction, MAPK signaling pathway, IL—17 signaling pathway, fluid shear stress and atherosclerosis and lipid and atherosclerosis were the top 5 significant pathways. The MAPK pathway is a well-known signaling pathway that is closely associated with the development and progression of various tumors^25,26^. A previous study showed that RAS or BRAF mutations were detected in approximately 32% of all Barrett’s adenocarcinomas, which indicated that disruption of the MAPK kinase pathway is a frequent but also early event in the development of Barrett’s adenocarcinoma^27^.

CD1a is a surface glycoprotein of 43–49 kDa that has been shown to be expressed by immune cells such as dendritic cells and Langerhans cells^28^. Cappello et al. proposed that CD1a could be expressed in metaplastic epithelium of Barrett’s esophagus, including gastric and intestinal types. However, normal gastrointestinal tissues do not express CD1a^28^. In BE tissues, metaplastic epithelial cells expressing higher CD1a levels could help distinguish gastric-type Barrett’s metaplasia from the presence of ectopic gastric epithelium in the esophageal mucosa^28^. Moreover, epithelial CD1a + cells may interact with dendritic cells or T cells in the development of BE^28^.Fatty acid binding protein 4 (FABP4), predominantly expressed in adipocytes and macrophages, is associated with the development and progression of various kinds of tumors^29^.FABP4 may have a potential associations with hyperlipidemia, hyperinsulinemia, and insulin resistance, indirectly affecting cancer cells by influencing these factors^30^. Multiple effects mediated by FABP4, such as insulin resistance, promote BE carcinogenesis^31^.

Lactotransferrin (LTF) is a member of the transferrin family that transfers iron to cells and controls the levels of free iron in the blood and external secretions. Some studies have reported that LTF is significantly lower in tumor tissues and that LTF may have a potential role in suppressing tumor growth and development^32,33^. For patients with papillary thyroid carcinoma, macrophages, mast cells, natural killer (NK) cells, Tfh cells, activated dendritic cells (aDCs), B cells, Tregs, CD8 + T cells and DCs were associated with LTF expression, which indicated that LTF plays an important role in the tumor microenvironment^32^. Insulin receptor (INSR) is a proliferation regulator involved in aggressive behaviors in many types of cancer^34^.In patients with BE, the insulin/insulin-like growth factor axis can mediate cancer progression and cause hyperinsulinemia and insulin resistance. The specific mechanisms involved in this tumor-promoting activity are unclear^31^. In gastric cancer, the high expression of INSR correlates with HER2 status and may have putative therapeutic implications^35^. In addition, pepsinogen C (PGC)is expressed only in the mucosa of the gastric fundus and is expressed by all regions of the gastric mucosa. In a preliminary study, PGC was expressed by columnar non goblet cells in most areas without specialized intestinal metaplasia which can help clinicians identify high risk BE patients and guide endoscopic surveillance^36^. Semaphorin 3C (SEMA3C) has been reported to drive a number of oncogenic programs, correlate poor cancer prognosis, and promote the progression of multiple different cancer types^37,38^.

The transcription factor 7-like 2 (TCF7L2) gene has been identified as a novel transcription factor involved in epithelial-mesenchymal transition (EMT) in tumor cells^39^. TCF7L2 is a member of the Wnt/b-catenin signaling pathway, which plays an important role in metabolism, cell differentiation/proliferation, and cell death^39^. The Wnt − /β-catenin signaling pathway is responsible for cell growth, motility and differentiation during embryogenesis. The Wnt/b-catenin signaling pathway has been reported to be activated in the progression of BE^40^. However the specific role of TCF7L2 in the development of BE is not fully understood. Previous studies have shown that TCG7L2 is closely associated with the development, progression and distant metastasis of various cancers such as pancreatic cancer^41^. The overexpressed TCF7L2 was associated with poor overall survival in patients with glioma^42^.

Finally, to explore the diagnostic accuracy of key genes, we performed ROC curve analysis and found a strong accuracy for the INSR (AUC = 0.93), which also provides a new biomarker for the diagnosis of BE.

Multiple analyses were performed to systematically investigate immune related genes, immune cells and their relationships in BE patients. Our study still has some limitations due to a lack of experimental validation and the use of a single dataset from the GEO. Future research needs to explore the detailed mechanism between the expression of distinct biomarkers and BE.

## Conclusions

In our present study, we identified 7 key genes, CD1A, LTF, FABP4,PGC, TCF7L2, INSR and SEMA3C as potential immune-related biomarkers in BE. Our study reveals the association between immune related genes and immune cells in BE patients for the first time. Our findings improve the understanding of the molecular mechanisms in BE and provide suggestions for novel therapy and diagnostic methods for BE patients.

## Supplementary Information

Supplementary Information 1.

Supplementary Information 2.

Supplementary Information 3.

## Data Availability

The raw data of this study are derived from the GEO data portal(https://www.ncbi.nlm.nih.gov/geo/), which are publicly available databases.
